# Clinical and molecular delineation of classical-like Ehlers–Danlos syndrome through a comprehensive next-generation sequencing-based screening system

**DOI:** 10.3389/fgene.2023.1234804

**Published:** 2023-08-30

**Authors:** Tomomi Yamaguchi, Kazuo Yamada, So Nagai, Toshiya Nishikubo, Norimichi Koitabashi, Masako Minami-Hori, Masaaki Matsushima, Yuka Shibata, Hiroki Ishiguro, Hiromi Sanai, Tomomi Fujikawa, Yuri Takiguchi, Ken-Ichi Matsumoto, Tomoki Kosho

**Affiliations:** ^1^ Center for Medical Genetics, Shinshu University Hospital, Matsumoto, Japan; ^2^ Department of Medical Genetics, Shinshu University School of Medicine, Matsumoto, Japan; ^3^ Division of Clinical Sequencing, Shinshu University School of Medicine, Matsumoto, Japan; ^4^ Department of Biosignaling and Radioisotope Experiment, Interdisciplinary Center for Science Research, Head Office for Research and Academic Information, Shimane University, Izumo, Japan; ^5^ Department of Legal Medicine, Faculty of Medicine, Shimane University, Izumo, Japan; ^6^ Problem-Solving Oriented Training Program for Advanced Medical Personnel: NGSD (Next-Generation Super Doctor) Project, Matsumoto, Japan; ^7^ Division of Neonatal Intensive Care, Nara Medical University, Nara, Japan; ^8^ Department of Cardiovascular Medicine, Gunma University Graduate School of Medicine, Maebashi, Japan; ^9^ Division of Dermatology, Asahikawa City Hospital, Asahikawa, Japan; ^10^ Department of Neurology, Faculty of Medicine and Graduate School of Medicine, Hokkaido University, Sapporo, Japan; ^11^ Division of Clinical Genetics, Hokkaido University Hospital, Sapporo, Japan; ^12^ Department of Clinical Genetics, Graduate School of Medicine, University of Yamanashi, Chuo, Japan; ^13^ Department of Obstetrics and Gynecology, Yamaguchi Prefectural Grand Medical Center, Yamaguchi, Japan; ^14^ Department of Medical Genetics, Yamaguchi Prefectural Grand Medical Center, Yamaguchi, Japan; ^15^ Research Center for Supports to Advanced Science, Shinshu University, Matsumoto, Japan

**Keywords:** Ehlers-Danlos syndrome, classical-like, *TNXB*, tenascin-X, connective tissue disorder

## Abstract

Classical-like Ehlers–Danlos syndrome (clEDS) is an autosomal recessive disorder caused by complete absence of tenascin-X resulting from biallelic variation in *TNXB*. Thus far, 50 patients from 43 families with biallelic *TNXB* variants have been identified. Accurate detection of *TNXB* variants is challenging because of the presence of the pseudogene *TNXA*, which can undergo non-allelic homologous recombination. Therefore, we designed a genetic screening system that is performed using similar operations to other next-generation sequencing (NGS) panel analyses and can be applied to accurately detect *TNXB* variants and the recombination of *TNXA*-derived sequences into *TNXB*. Using this system, we identified biallelic *TNXB* variants in nine unrelated clEDS patients. *TNXA*-derived variations were found in >75% of the current cohort, comparable to previous reports. The current cohort generally exhibited similar clinical features to patients in previous reports, but had a higher frequency of gastrointestinal complications (e.g., perforation, diverticulitis, gastrointestinal bleeding, intestinal obstruction, rectal/anal prolapse, and gallstones). This report is the first to apply an NGS-based screening for *TNXB* variants and represents the third largest cohort of clEDS, highlighting the importance of increasing awareness of the risk of gastrointestinal complications.

## Introduction

The Ehlers–Danlos syndromes (EDSs) are a group of hereditary connective tissue disorders (HCTDs) characterized by skin hyperextensibility, joint hypermobility, and tissue fragility. They are classified into 14 subtypes based on symptoms and causative genes according to the 2017 International Classification (Malfait et al., 2017) and subsequent findings (Malfait et al., 2017). The classical-like type of EDS (clEDS) is an autosomal recessive disorder caused by complete absence of tenascin-X (TNX) resulting from biallelic variation in *TNXB*. The major criteria of clEDS include: 1) skin hyperextensibility, with velvety skin texture and absence of atrophic scarring; 2) generalized joint hypermobility, with or without recurrent dislocations; and 3) easily bruisable skin/spontaneous ecchymosis. The minor criteria include: 1) foot deformities, including broad/plump forefeet, brachydactyly with excessive skin, pes planus, hallux valgus, and piezogenic papules; 2) edema in the legs in the absence of cardiac failure; 3) mild proximal and distal muscle weakness; 4) axonal polyneuropathy; 5) atrophy of muscles in the hands and feet; 6) acrogeric hands, mallet finger(s), clinodactyly, and brachydactyly; and 7) vaginal/uteral/rectal prolapse (Brady et al., 2017; Malfait et al., 2017).

**FIGURE 1:**
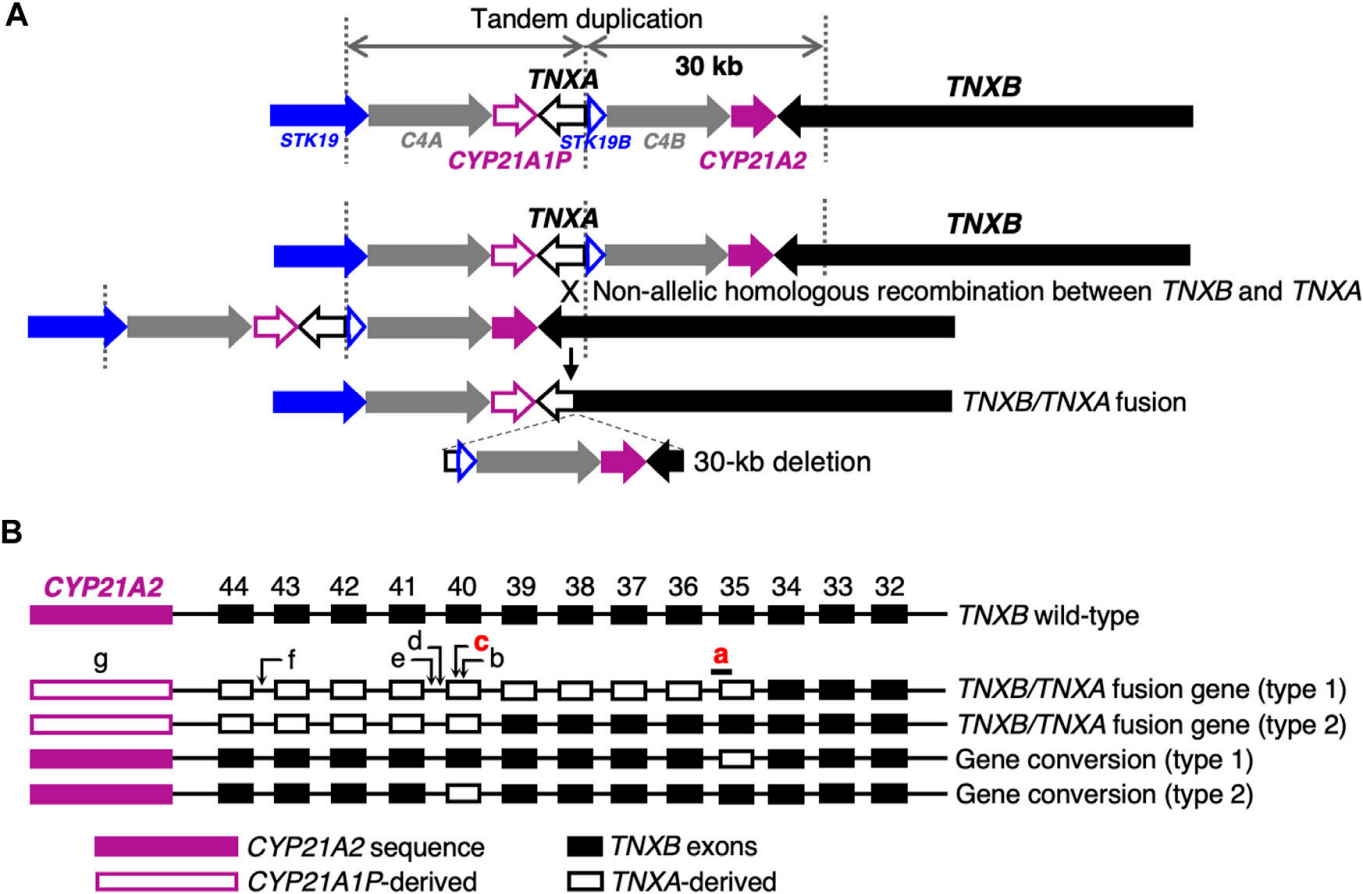
Schematic diagram of *TNXB/TNXA* fusion genes. **(A)** *TNXB/TNXA* fusion genes resulting from non-allelic homologous recombination between the *TNXA* pseudogene and *TNXB*, resulting in 30-kb deletions. **(B)** *TNXB/TNXA* fusion genes (type 1 and type 2) and gene conversions (type 1 and type 2). Seven indices of *TNXA*-derived variations are shown: **a**, 120-bp deletion (c.11435_11524 + 30del) in exon 35–intron 35; **b**, c.12150C>G,p.(Arg4050 = ) in exon 40; **c**, c.12174C>G,p.(Cys4058Trp) in exon 40; **d**, c.12204 + 39dup in intron 40; **e**, c.12204 + 43T>G in intron 40; **f**, c.12628-52A>G in intron 43; **g**, *CYP21A2* deletion. Only a 120-bp deletion in exon 35–intron 35 and c.12174C>G in exon 40 shown in red have been reported to be pathogenic (Demirdas et al., 2017).

Next-generation sequencing (NGS) panel-based genetic screening is a useful approach for differentiating multiple subtypes of EDS and other HCTDs with overlapping clinical manifestations (Yamaguchi et al., 2023). However, the presence of the pseudogene *TNXA*, which is >97% identical to the 3′end of *TNXB* (exons 32–44), makes it challenging to detect *TNXB* variants by conventional NGS analysis (Demirdas et al., 2017). *TNXA* is located approximately 30 kb centromere-proximal to *TNXB*. The intervening 30-kb unit is duplicated in tandem, and can undergo non-allelic homologous recombination, resulting in 30-kb deletions that include the 3′end of *TNXB* or gene conversions (Figures 1A, B) (Morissette et al., 2015; Demirdas et al., 2017). A 120-bp deletion of *TNXB* exon 35–intron 35 (c.11435_11524 + 30del) (Figure 1B) and c.12174C>G,p.(Cys4058Trp) in exon 40 (Figure 1B) are indices of *TNXA*-derived variations (Morissette et al., 2015), and have been determined to be pathogenic (Demirdas et al., 2017).

Fifty patients from 43 families with biallelic *TNXB* variants have been identified (Burch et al., 1997; Schalkwijk et al., 2001; Voermans et al., 2009; Hendriks et al., 2012; Pénisson-Besnier et al., 2013; Sakiyama et al., 2015; Chen et al., 2016; Demirdas et al., 2017; Micale et al., 2019; Rymen et al., 2019; Brisset et al., 2020; Green et al., 2020; Colman et al., 2021; Watanabe et al., 2021; Al-Harbi et al., 2022; Santoreneos et al., 2022). The largest cohort of clEDS (20 patients) was reported by Green et al. (2020), and the second largest was reported by Demirdas et al. (2017), which comprised 11 patients from seven families previously reported by the same authors (Schalkwijk et al., 2001; Voermans et al., 2009; Hendriks et al., 2012) and six new patients from four families. Genetic testing of clEDS has been performed by Sanger sequencing using *TNXB*-specific primers, Sanger sequencing following long PCR using *TNXB*-specific primers for exons 32–44, and Sanger sequencing or NGS for other regions.

We describe here the development of a genetic screening system for the accurate detection of *TNXB* variants and *TNXA*-derived sequences recombined into *TNXB* that was designed to be performed similarly to other NGS panel analyses. This is also the third largest cohort and the first report of an Asian cohort of clEDS patients (eight Japanese; one Chinese), all of whom were found to have biallelic *TNXB* variation.

## Materials and methods

### Ethics statement

This study was approved by the Ethics Committee of Shinshu University School of Medicine (Nos. 435 and 628). Written consent was obtained from all participants before joining the study.

### NGS panel

An NGS panel of 53 genes associated with various HCTDs, including *TNXB* and pseudogene *TNXA* (Supplementary Table S1), was designed by Ion AmpliSeq Designer (https://ampliseq.com/browse.action). *TNXA* was included for detection of *TNXA*-derived sequences recombined into *TNXB.*

### Standard NGS panel analysis

Genomic DNA was extracted from peripheral blood using a QIAamp DNA Blood Mini Kit on a QIAcube (Qiagen, Valencia, CA, USA). Library preparation was performed using an Ion AmpliSeq Library Kit Plus (Thermo Fisher Scientific, Waltham, MA, USA). Sequencing was performed on an Ion Torrent system (Ion Chef and Ion GeneStudio S5) using an Ion 510 & 520 & 530 Kit—Chef and an Ion 520 Chip Kit (Thermo Fisher Scientific). Sequencing data were mapped using Torrent Suite software (Thermo Fisher Scientific) to human genome hg19, which masked exons 32–44 of *TNXB* and *TNXA* sequence by replacing them with “N”s. Single-nucleotide variants (SNVs) and small insertions/deletions were detected from the mapped data using the Torrent Variant Caller plug-in. Copy number variation (CNV) was analyzed using the CNV visualization method for amplification-based NGS data that was established by Nishio et al. (2018). The variants in *TNXB* were described using the NM_019105.6 transcript reference sequence, and the variant nomenclature was in accordance with the Human Genome Variation Society recommendations.

### Modified NGS panel analysis using long PCR-amplified product

The method for library preparation of exons 32–44 of *TNXB* was identical to the standard NGS panel analysis, except that the template DNA was a long PCR product (diluted with water 1:200) amplified using the primer set reported by Micale et al. (2019). The forward primer, TNXB-ex31-F (5′-GTC​TCT​GCC​CTG​GGA​ATG​A-3′; described as TNXB-LongPCR-F in Micale et al. (2019)), is a *TNXB*-specific sequence from *TNXB* exon 31, and the reverse primer, TNXB-ex44-R (5′-TGT​AAA​CAC​AGT​GCT​GCG​A-3′; described as TNXB-LongPCR-R in Micale et al. (2019)), was designed based on a common sequence of *TNXB* and *TNXA*, which is useful because it can also detect recombinant alleles. Library preparation was performed simultaneously with the standard NGS panel analysis. Libraries prepared to a concentration of 100 pM by an Ion AmpliSeq Library Kit Plus were mixed to match the number of amplicons and sequenced. The *TNXA* sequence of the human genome hg19 was masked by replacement with “N”s. However, because it was difficult to align fragments containing *TNXA*-derived 120-bp deletions to exon 35–intron 35 of *TNXB,* an exon 35/intron 35-homologous reference sequence of *TNXA* was not replaced with “N”s, and the fragments containing the *TNXA*-derived 120-bp deletions were aligned on *TNXA*. Additionally, a hotspot file to be called with or without a variant included one within exon 35 of *TNXB* and one within the exon 35-homologous sequence of *TNXA* (Supplementary Table S2). In other words, if both exon 35 of *TNXB* and the exon 35-homologous sequence of *TNXA* are called, the target is heterozygous for a *TNXA*-derived 120-bp deletion, whereas if exon 35 of *TNXB* is unable to produce a result because of lack of coverage (no call) and the exon 35-homologous sequence of *TNXA* is called, the target is homozygous for the *TNXA*-derived 120-bp deletions. A hotspot file also included c.12150C>G,p.(Arg4050 = ) and c.12174C>G,p.(Cys4058Trp) in exon 40, c.12204 + 39dup and c.12204 + 43T>G in intron 40, and c.12628-52A>G in intron 43 as indices of the *TNXA*-derived variations (Figure 1B and Supplementary Table S2).

### Sanger sequencing

SNVs and small deletions were confirmed by Sanger sequencing, which was performed on a 3500 Genetic Analyzer using a BigDye Direct Cycle Sequencing Kit with M13 tailed primers and a BigDye XTerminator Purification Kit (Thermo Fisher Scientific), according to the manufacturer’s instructions. For SNVs and small deletions in exons 32–44 of *TNXB*, sequencing was performed using a long PCR-amplified product (diluted with water 1:50) as template DNA.

### Phasing analysis

For Patient 6, long PCR was performed using primers TNXB-ex35-F (5′-AAA​CTC​CAG​GGG​CTG​ATC​C-3′), which binds specifically to normal exon 35 of *TNXB*, and TNXB-ex44-R, which binds to both *TNXB* and *TNXA*. Nested PCR of the region around exon 40 was performed using the long PCR-amplified product (diluted with water 1:50) as template DNA. For Patient 9, long PCR was performed using primers TNXB-ex26-F (5′-TGT​GGG​TGT​GAC​AGG​TGA​GT-3′) and TNXB-ex35-R (5′-TGG​TGA​GGA​AGC​CTG​TGA​GA-3′), which bind specifically to normal exon 35 of *TNXB*. Nested PCR of the region around exon 27 was performed using the long PCR-amplified product (diluted with water 1:50) as template DNA. Sanger sequencing was performed as described above.

### Multiplex ligation-dependent probe amplification (MLPA) analysis

CNVs were confirmed by MLPA using a SALSA MLPA Kit P155-D2 (MRC-Holland, Amsterdam, Netherlands) for *TNXB* and *CYP21A2*. *CYP21A2* is a causative gene for autosomal recessive congenital adrenal hyperplasia due to 21-hydroxylase deficiency. MLPA analysis for *CYP21A2* was performed to confirm the presence of a 30-kb deletion, not to reveal the carrier status of autosomal recessive congenital adrenal hyperplasia. Electrophoresis was conducted on a 3500 Genetic Analyzer (Thermo Fisher Scientific) and the data were analyzed with Coffalyzer.Net (MRC-Holland).

### Western blotting

Blood samples were centrifuged to separate serum and frozen at −80°C until use. Commercially available human sera (Lonza; BioWhittaker, Walkersville, MD, USA) were also used as a normal control. Western blotting was performed as described previously (Yamada et al., 2016).

### Nano-liquid chromatography tandem mass spectrometry (nano-LC/MS/MS)

Blood samples were centrifuged to separate serum and frozen at −80°C until use. Commercially available human sera (Lonza; BioWhittaker, Walkersville, MD, USA) were also used as a normal control. Measurement of the serum form of TNX (sTNX) concentration with AVAVSGLDPAR peptide was performed by using a quantitative nano-LC/MS/MS method as described previously (Yamada et al., 2016). The sTNX concentrations in each sample were measured three times and triplicate experiments were performed. Data are expressed as means ± standard error. The mass spectrometry proteomics data have been deposited to the ProteomeXchange Consortium via the PRIDE (Perez-Riverol et al., 2022) partner repository with the dataset identifier PXD043691.

## Results

**FIGURE 2:**
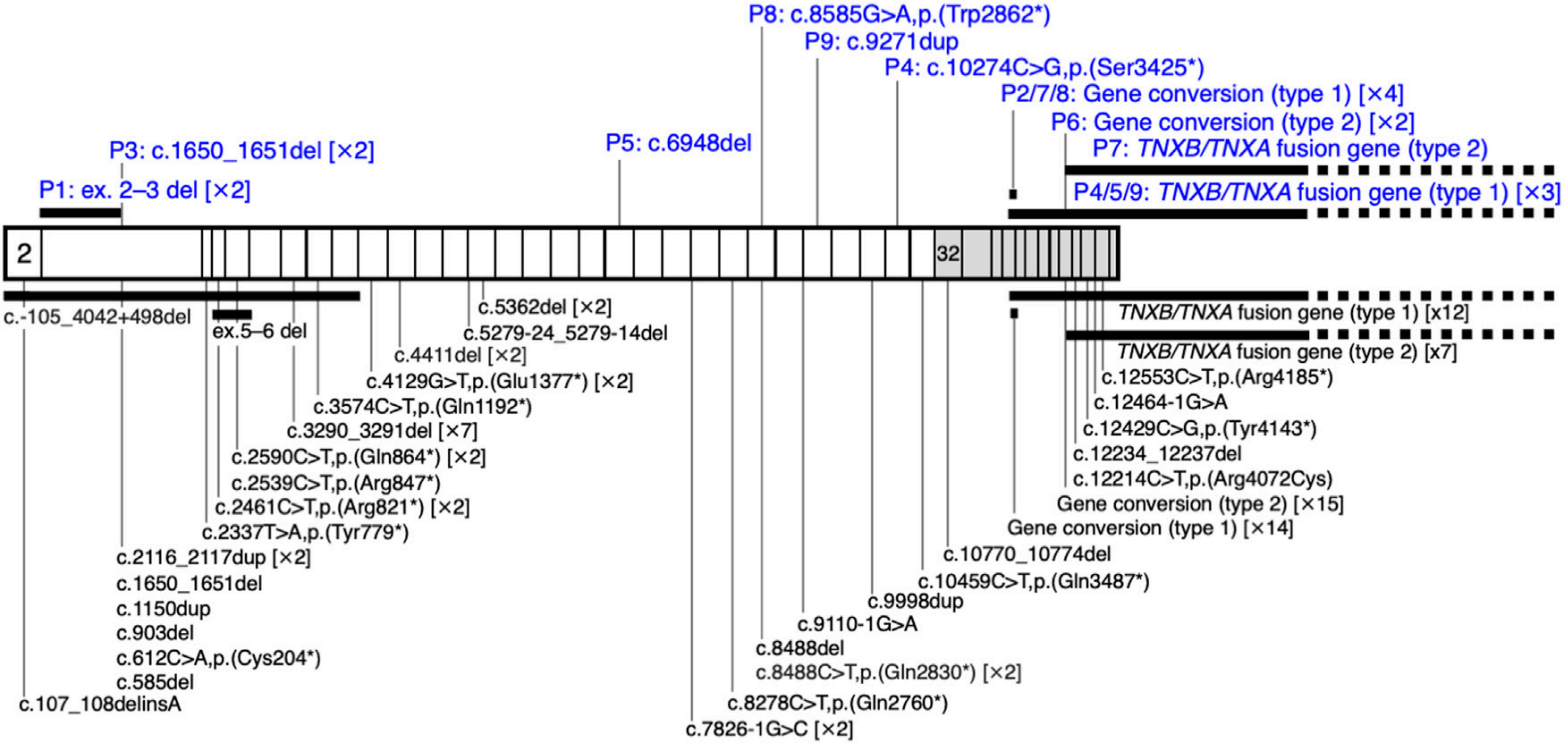
Schematic representation of the distribution of *TNXB* variants in a cohort of nine patients with clEDS. Illustration of *TNXB* variants based on NM_019105.6 of the NCBI reference sequence database (https://www.ncbi.nlm.nih.gov/RefSeq/). Exons 32–44 are highlighted with gray boxes. Variants found in the current study of patients (P)1–9 are shown in blue, above the mRNA transcript. Previously reported variants (Burch et al., 1997; Schalkwijk et al., 2001; Voermans et al., 2009; Hendriks et al., 2012; Pénisson-Besnier et al., 2013; Sakiyama et al., 2015; Chen et al., 2016; Demirdas et al., 2017; Micale et al., 2019; Rymen et al., 2019; Brisset et al., 2020; Green et al., 2020; Colman et al., 2021; Watanabe et al., 2021; Al-Harbi et al., 2022; Santoreneos et al., 2022) are shown below the mRNA transcript.

Nine unrelated patients with clEDS were found to have homozygous or compound heterozygous pathogenic variants in *TNXB* (Table 1). The variants for each allele were detected as follows: standard NGS panel analysis in Patients 1 and 3; modified NGS panel analysis using long PCR-amplified product in Patients 2, 6, and 7; and standard NGS panel analysis and modified NGS panel analysis using long PCR-amplified product in Patients 4, 5, 8, and 9. Results of modified NGS panel analysis using long PCR-amplified product were identical to those of Sanger sequencing or MLPA. Clinical and molecular features of the patient cohort are summarized in Table 2 and Figure 2, and detailed descriptions of each case are provided below.

**TABLE 1:** Nine unrelated clEDS patients with homozygous or compound heterozygous pathogenic variants in *TNXB* (NM_019105.6).

Patient No.	Sex	Age at last observation (years)	Allele 1	Allele 2	Serum Tenascin-X
1	Female	60	Exons 2–3 deletion	Exons 2–3 deletion	N/A
2	Male	65	Gene conversion (type 1) including c.11435_11524 + 30del	Gene conversion (type 1) including c.11435_11524 + 30del	Absence
Son	Male	32	Gene conversion (type 1) including c.11435_11524 + 30del	−	N/A
3	Male	d.62	c.1650_1651del,p.(Glu552Argfs*41)	c.1650_1651del,p.(Glu552Argfs*41)	N/A
Daughter	Female	21	c.1650_1651del,p.(Glu552Argfs*41)	−	N/A
4	Female	27	c.10274C>G,p.(Ser3425*)	*TNXB/TNXA* fusion gene (type 1) including c.11435_11524 + 30del and c.12174C>G	N/A
Father	Male	N/A	c.10274C>G,p.(Ser3425*)	−	N/A
Mother	Female	N/A	−	*TNXB/TNXA* fusion gene (type 1) including c.11435_11524 + 30del and c.12174C>G	N/A
Brother	Male	N/A	−	*TNXB/TNXA* fusion gene (type 1) including c.11435_11524 + 30del and c.12174C>G	N/A
5	Female	47	c.6948del,p.(Asp2317Thrfs*53)	*TNXB/TNXA* fusion gene (type 1) including c.11435_11524 + 30del and c.12174C>G	Absence
Father	Male	77	−	*TNXB/TNXA* fusion gene (type 1) including c.11435_11524 + 30del and c.12174C>G	Reduction
Mother	Female	75	c.6948del,p.(Asp2317Thrfs*53)	−	Reduction
Sister	Female	52	−	*TNXB/TNXA* fusion gene (type 1) including c.11435_11524 + 30del and c.12174C>G	Reduction
6	Female	59	Gene conversion (type 2) including c.12174C>G	Gene conversion (type 2) including c.12174C>G	Absence
7	Male	61	Gene conversion (type 1) including c.11435_11524 + 30del	*TNXB/TNXA* fusion gene (type 2) including c.12174C>G	Absence
8	Female	8	c.8585G>A,p.(Trp2862*)	Gene conversion (type 1) including c.11435_11524 + 30del	N/A
Father	Male	48	−	Gene conversion (type 1) including c.11435_11524 + 30del	N/A
Mother	Female	48	c.8585G>A,p.(Trp2862*)	−	N/A
9	Female	69	c.9271dup,p.(Gln3091Profs*31)	*TNXB/TNXA* fusion gene (type 1) including c.11435_11524 + 30del and c.12174C>G	N/A

d, Died; N/A, not available.

**TABLE 2:** Detailed clinical features of the current cohort of patients with clEDS.

Patient No.	1	2	3	4	5	6	7	8	9	
Age at last observation (years)	60	65	d.62	27	47	59	61	8	69	
Sex	Female	Male	Male	Female	Female	Female	Male	Female	Female	
Ethnicity	Japanese	Japanese	Japanese	Japanese	Japanese	Chinese	Japanese	Japanese	Japanese	
Skin hyperextensibility	+	−	N/A	+	+	+	+	+	+	7/8 (87.5%)
Velvety skin texture	+	−	N/A	+	−	N/A	+	+	−	4/7 (57.1%)
Atrophic scarring	+	−	N/A	N/A	+	+	−	−	+	4/7 (57.1%)
Generalized joint hypermobility	+	− (only fingers and elbows)	N/A	+	+	+	− (only elbows and knees)	+	−	5/8 (62.5%)
Beighton score	N/A	N/A	N/A	N/A	6/9	N/A	4/9	9/9	2/9	
Recurrent dislocations	+	−	N/A	+(shoulder)	+	+(shoulder, finger, hip, toe)	+(shoulder)	+(finger)	+(shoulder, toe)	7/8 (87.5%)
Easily bruisable skin/spontaneous ecchymosis	+	+	N/A	+	+	+	+	+	+	8/8 (100%)
Eye bleeding	N/A	+(subconjunctiva)	N/A	N/A	+(conjunctiva)	N/A	N/A	+(sclera)	N/A	
Hand deformities	Acrogeric hands, mallet fingers, clinodactyly, brachydactyly	Acrogeric hands, brachydactyly	N/A	−	Acrogeric hands	N/A	N/A	Mallet fingers, brachydactyly	Acrogeric hands, clinodactyly, brachydactyly	5/6 (83.3%)
Foot deformities	Broad/plump forefoot, brachydactyly with excessive skin, pes planus, hallux valgus, piezogenic papules	Broad/plump forefoot, pes planus	N/A	−	Pes planus, hallux valgus	N/A	Pes planus	Broad/plump forefoot, pes planus, piezogenic papules	Broad/plump forefoot, brachydactyly with excessive skin, pes planus	6/7 (85.7%)
Skeletal features	NA	N/A	N/A	N/A	N/A	N/A	Pectus excavatum, scoliosis	Fractures	N/A	
Muscle weakness	+	N/A	N/A	−	+	N/A	N/A	−	+	3/5 (60.0%)
Muscle Atrophy	+	N/A	N/A	−	+	N/A	N/A	−	−	2/5 (40.0%)
Other musculoskeletal features	Distal inter-phalangeal joint pain	Muscle deficiencies	N/A	Meniscus injury, anterior cruciate ligament injury	N/A	N/A	N/A	N/A	N/A	
Cardiovascular abnormalities	N/A	Tetralogy of Fallot	N/A	−	Chronic heart failure, mitral regurgitation	Subcutaneous hematoma	Angina pectoris	Mitral regurgitation	Subcutaneous hematoma	
Hematoma	N/A	N/A	N/A	N/A	N/A	Subcutaneous, rectal	N/A	N/A	Subcutaneous	
Gastrointestinal features	Reflux esophagitis, multiple gastric polyps, low-grade tubular adenoma	Recurrent intestinal obstruction, colonic diverticulitis, rectal prolapse, gallstones, constipation	Intestinal obstruction	N/A	Small bowel diverticula and perforation, constipation, vomiting and diarrhea after eating	Anal prolapse, anal laceration, gallstones, appendicitis	Small bowl perforation, esophageal perforation, diverticulitis	Anal prolapse	Small bowl perforation, rectal prolapse, gallstones	8/8 (100%)
Urogenital features	N/A	−	N/A	N/A	N/A	N/A	Distended bladder, bladder diverticula, difficulty urinating	−	Difficulty urinating due to vaginal prolapse	
Hernia	N/A	−	N/A	N/A	N/A	N/A	Umbilical, inguinal	−	Abdominal wall incisional hernia after diaphragmatic hernia repair, hiatal hernia	
Others	Edema in the legs, axonal polyneuropathy	Translucent skin, skin striae, gingival recession, mandibular protrusion, migraine, hemopneumothrrax	N/A	N/A	Congenital cataract, generalized pain	Generalized and wandering pain, anxiety disorder, massive swelling after insect bites	Translucent skin	Stomatitis, peripheral cyanotic skin in cool environments, hyperventilation	Pulmonary embolism following gallbladder surgery, early Parkinson’s disease	

d, died; N/A, not available.

### Patient 1

Patient 1 is a 60-year-old Japanese woman who was referred to us as a suspected case of clEDS because of skin hyperextensibility, fragility, and bruisability of the skin, as well as joint hypermobility and recurrent dislocation. She had gastrointestinal complications, including reflux esophagitis, multiple gastric polyps, and colonic polyp(s), diagnosed as low-grade tubular adenomas. She presented with numbness in the hands and feet, suggestive of neuropathy.

**FIGURE 3:**
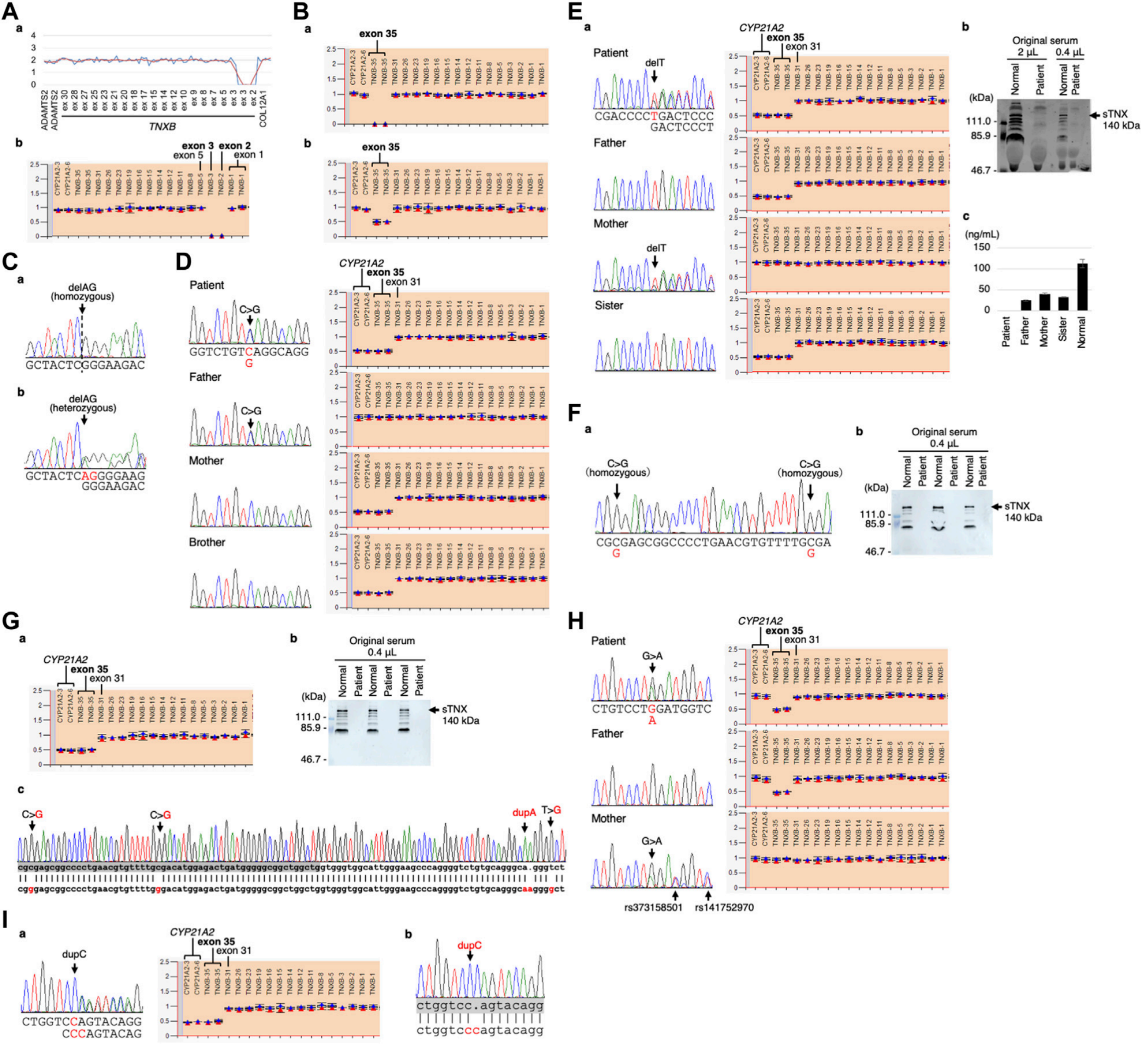
Molecular investigation of Patients 1–9. **(A)** Patient 1: homozygosity for exons 2–3 deletion detected by the CNV visualization method for an amplification-based NGS data **(a)** and validated by MLPA **(b)**. **(B)** Patient 2: homozygosity for a gene conversion characterized by *TNXA*-derived variation with validation of the exon 35 deletion by MLPA in the patient **(a)** and his son **(b)**. **(C)** Patient 3: homozygosity for a frameshift variant c.1650_1651del,p.(Glu552Argfs*41) validated by Sanger sequencing in the patient **(a)** and his daughter **(b)**. **(D)** Patient 4: compound heterozygosity for a nonsense variant c.10274C>G,p.(Ser3425*) and a *TNXB/TNXA* fusion gene characterized by *TNXA*-derived variation with validation of the nonsense variant by Sanger sequencing (left) and the *TNXB* exon 35 deletion and *CYP21A2* deletion by MLPA (right) in the patient and her family. **(E)** Patient 5: compound heterozygosity for a frameshift variant c.6948del,p.(Asp2317Thrfs*53) and a *TNXB/TNXA* fusion gene characterized by *TNXA*-derived variation with validation of the frameshift variant by Sanger sequencing (left) and the *TNXB* exon 35 deletion and *CYP21A2* deletion by MLPA (right) in the patient and her family **(a)**. Western blot analysis of sTNX in the patient **(b)** and quantification of sTNX in the patient and her family using nano-LC/MS/MS **(c)**. **(F)** Patient 6: homozygosity for a gene conversion characterized by *TNXA*-derived variation with validation by Sanger sequencing **(a)** and triplicate Western blot analysis of sTNX in the patient **(b)**. **(G)** Patient 7: compound heterozygosity for a gene conversion and a *TNXB/TNXA* fusion gene characterized by *TNXA*-derived variation with validation of the *TNXB* exon 35 deletion and *CYP21A2* deletion by MLPA **(a)**. Triplicate Western blot analysis of sTNX **(b)**. Sanger sequencing of the region around exon 40 in the normal exon 35 allele **(c)**, with the upper row showing the *TNXB* sequence and the lower row showing the *TNXA* sequence. *TNXB* exon 40 is highlighted with a gray box. **(H)** Patient 8: compound heterozygosity for a nonsense variant c.8585G>A,p.(Trp2862*) and a gene conversion characterized by *TNXA*-derived variation with validation of the nonsense variant by Sanger sequencing (left) and the *TNXB* exon 35 deletion by MLPA (right) in the patient and her parents. **(I)** Patient 9: compound heterozygosity for a frameshift variant c.9271dup,p.(Gln3091Profs*31) and a *TNXB/TNXA* fusion gene characterized by *TNXA*-derived variation with validation of the frameshift variant by Sanger sequencing (left) and the *TNXB* exon 35 deletion and *CYP21A2* deletion by MLPA (right) **(a)**. Sanger sequencing of the frameshift variant c.9271dup,p.(Gln3091Profs*31) in the normal exon 35 allele **(b)**, with the upper row showing the *TNXB* sequence and the lower row showing the *TNXA* sequence. *TNXB* exon 27 is highlighted with a gray box.

The CNV visualization method for amplification-based NGS data (Nishio et al., 2018) revealed two copy losses spanning the start of exon 2 to the middle of exon 3 (Figure 3Aa). The start codon is in exon 2. The deletion breakpoints are unknown. MLPA also showed the two copy losses of exons 2 and 3 (Figure 3Ab).

### Patient 2

**FIGURE 4:**
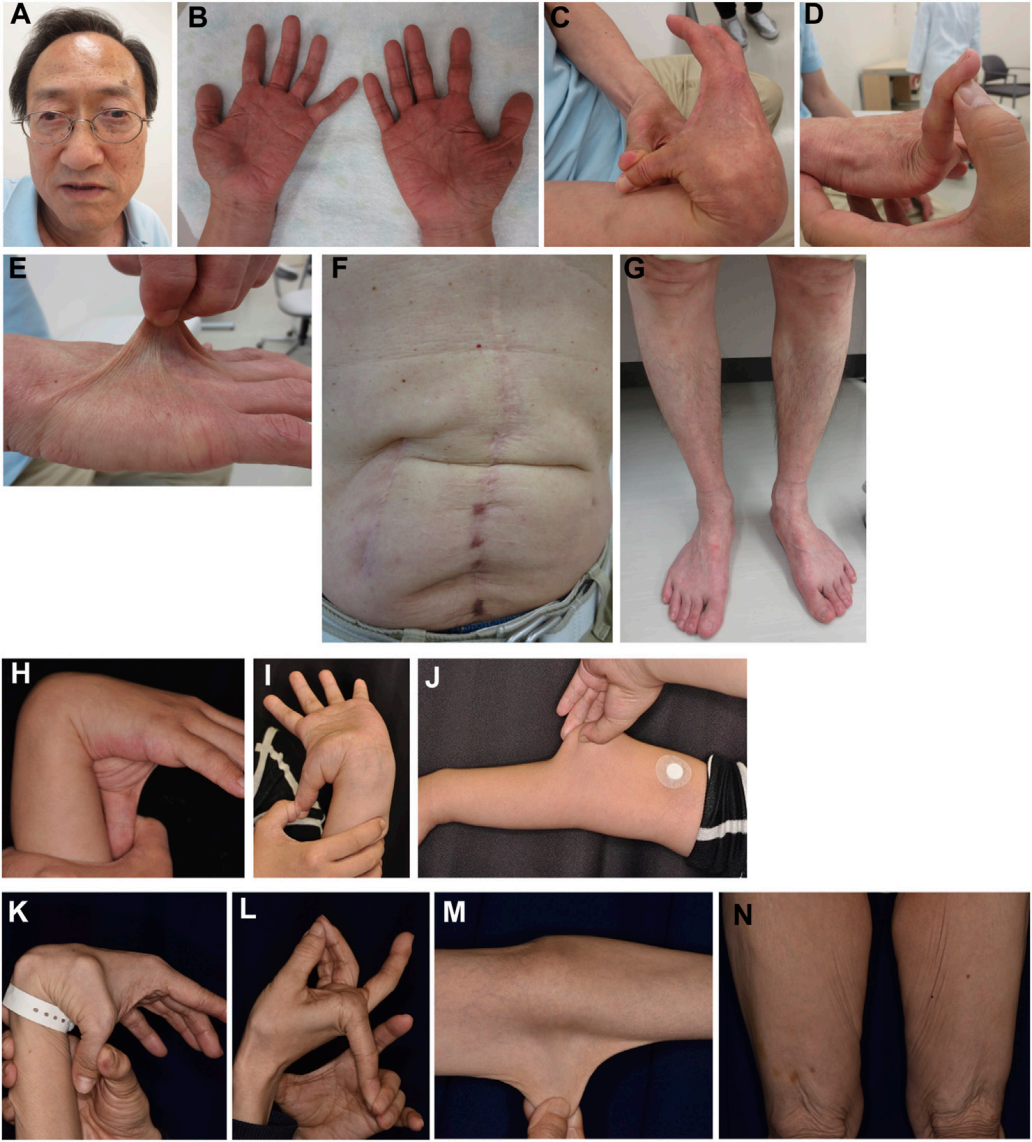
Clinical photographs of Patients 2, 4, and 5. **(A–G)** Patient 2 at age 65 years, showing jaw protrusion **(A)**, hands with thick fingers and wrinkled palms **(B)**, hypermobile finger joints **(C,D)**, hyperextensible skin **(E)**, atrophic scars at surgical sutures **(F)**, and valgus/flat feet **(G)**. **(H–J)** Patient 4 at age 27 years, showing finger joint hypermobility **(H,I)** and skin hyperextensiblity **(J)**. **(K–N)** Patient 5 at age 47 years, showing marked finger joint hypermobility **(K,L)** and skin hyperextensibility **(M)** and redundancy **(N)**.

Patient 2 is a 65-year-old Japanese man with tetralogy of Fallot that was surgically corrected at age 12 years. Subconjunctival hemorrhages and migraines were recurrent from age 28 years onwards. During open surgery for gallstones at age 34 years, he experienced excessive bleeding. At age 42 years, he developed bowel obstruction for which bowel resection surgery was complicated by difficulties in hemostasis and intestinal suture due to marked fragilities. Muscle defects in multiple organs were noted on histopathology. Pneumohemothorax occurred at age 44 years, which was treated with chest drainage followed by a thoracoscopic bullectomy, with no recurrence. Colonic diverticulitis occurred at age 47 years and was treated with antibiotics. He developed another bowel obstruction at age 54 years, for which bowel resection was performed. EDS was suspected on histopathology, and he was referred to our hospital for further assessment at age 56 years. His height was 162.4 cm (−1.4 standard deviation [SD]), weight was 51.3 kg (−1.1SD), and occipitofrontal circumference (OFC) was 56.2 cm (−0.9SD). He had jaw protrusion (Figure 4A), soft and wrinkled palms (Figure 4B), hypermobile and thick fingers (Figures 4C, D), skin hyperextensibility and translucency (Figure 4E), atrophic scars at surgical sutures (Figure 4F), valgus/flat feet with sole calluses (Figure 4G), skin striae, and gingival recession. His skin was thin and translucent, but not velvet-like. He was clinically suspected as having vascular EDS, and took alacepril to prevent arterial complications. His parents had no skin hyperextensibility or joint hypermobility. His son had generalized joint hypermobility in his preschool days, and a ligamental injury at the left ankle.

Modified NGS panel analysis using long PCR-amplified product detected a homozygous *TNXA*-derived 120-bp deletion in exon 35–intron 35 (c.11435_11524 + 30del). MLPA showed two copy losses of exon 35 in *TNXB* and a normal copy of *CYP21A2* (Figure 3Ba). Therefore, the patient was determined to have a type 1 gene conversion (Figure 1B). His son was found to have a one-copy loss of exon 35 in *TNXB* and a normal copy of *CYP21A2* (Figure 3Bb), confirming his carrier status for clEDS. Complete deficiency of sTNX in Patient 2 was detected through biochemical analysis using Western blotting (data not shown).

### Patient 3

Patient 3 was a Japanese man who died from tongue cancer at age 62 years. He developed intestinal obstruction at age 55, which required colostomy. His daughter had abnormal scarring, soft skin, and hypermobility of the finger joints. At age 21 years, she was referred to us for evaluation regarding EDS, and was suspected to have vascular EDS. She experienced three uncomplicated pregnancies and deliveries.

Standard NGS panel analysis revealed that Patient 3 had a homozygous frameshift variant c.1650_1651del,p.(Glu552Argfs*41) in exon 3, which was confirmed by Sanger sequencing (Figure 3Ca). His daughter was found to be heterozygous for the variant (Figure 3Cb), and had no other pathogenic variants in related genes, including *COL3A1*.

### Patient 4

Patient 4 is a 27-year-old Japanese woman who was referred to us at 32 weeks of gestation for suspected clEDS based on joint hypermobility (Figures 4H, I), hyperextensible, soft, and bruisable skin (Figure 4J), and recurrent dislocation. She had recurrent dislocation of the left shoulder, and underwent surgeries for meniscus injuries at ages 15 and 27 years. Cardiac ultrasonography detected no abnormalities.

Standard NGS panel analysis detected a heterozygous nonsense variant c.10274C>G,p.(Ser3425*) in exon 30, which was confirmed by Sanger sequencing (Figure 3D, left). Modified NGS panel analysis using long PCR-amplified product detected a heterozygous *TNXA*-derived 120-bp deletion in exon 35–intron 35 (c.11435_11524 + 30del), c.12150C>G,p.(Arg4050 = ) and c.12174C>G,p.(Cys4058Trp) in exon 40, c.12204 + 39dup and c.12204 + 43T>G in intron 40, and c.12628-52A>G in intron 43. The four variants in exon 40 and intron 40 were confirmed by Sanger sequencing (data not shown). MLPA showed a one-copy loss of exon 35 in *TNXB* and a one-copy loss of *CYP21A2* (Figure 3D, right). Sanger sequencing and MLPA analysis were also performed on her parents, which revealed the heterozygous nonsense variant in her father and the single copy loss of exon 35 in *TNXB* and *CYP21A2* in her mother (Figure 3D). Therefore, it was determined that Patient 4 was compound heterozygous for the nonsense variant and a type 1 *TNXB/TNXA* fusion gene (Figure 1B). Her brother was found to have a one-copy loss of exon 35 in *TNXB* and *CYP21A2* (Figure 3D), confirming his carrier status for clEDS.

### Patient 5

Patient 5 is a 47-year-old Japanese woman referred to us for a suspected diagnosis of EDS. She underwent surgery for congenital cataract. She was readily bruisable and experienced recurrent dislocation as a young child. At age 43 years, she suffered from recurrent intestinal perforation associated with multiple diverticula of the intestine. She has chronic heart failure associated with mitral valve regurgitation, generalized joint hypermobility (Beighton score 6/9) (Figures 4K, L), recurrent dislocation, skin hyperextensibility, redundancy, and bruisability (Figures 4M, N), conjunctival bruisability, generalized pain, and gastrointestinal symptoms (vomiting and diarrhea accompanied by intractable abdominal pain after eating). Her mother has bruisable skin, joint hypermobility with recurrent dislocation, pes planus, hallux valgus, and edema of the lower extremities. Her father and sister have no relevant features.

Standard NGS panel analysis detected a heterozygous frameshift variant c.6948del,p.(Asp2317Thrfs*53) in exon 20, which was confirmed by Sanger sequencing (Figures 3Ea, left). Modified NGS panel analysis using long PCR-amplified product detected a heterozygous *TNXA*-derived 120-bp deletion in exon 35–intron 35 (c.11435_11524 + 30del), c.12150C>G,p.(Arg4050 = ) and c.12174C>G,p.(Cys4058Trp) in exon 40, c.12204 + 39dup and c.12204 + 43T>G in intron 40, and c.12628-52A>G in intron 43. The four variants in exon 40 and intron 40 were confirmed by Sanger sequencing (data not shown). MLPA showed the one-copy loss of exon 35 in *TNXB* and a one copy loss of *CYP21A2* (Figure 3Ea, right). Sanger sequencing and MLPA analysis were performed on her parents, which revealed the heterozygous frameshift variant in her mother and the one-copy loss of exon 35 in *TNXB* and *CYP21A2* in her father (Figure 3Ea). Therefore, it was determined that Patient 5 was compound heterozygous for the frameshift variant and a type 1 *TNXB/TNXA* fusion gene (Figure 1B). Western blot analysis and nano-LC/MS/MS detection showed complete absence of sTNX in Patient 5 (Figures 3Eb,c). The mean sTNX concentration by nano-LC/MS/MS was 25 ± 2 ng/mL (22.1% of normal) in her father, 41 ± 1 ng/mL (36.3% of normal) in her mother, and 113 ± 10 ng/mL in the normal control (Figure 3Ec). Her sister was found to have the one-copy loss of exon 35 in *TNXB* and *CYP21A2,* and a mean sTNX concentration of 33 ± 1 ng/mL (29.2% of normal) (Figure 3Ec), confirming her carrier status for clEDS.

### Patient 6

**FIGURE 5:**
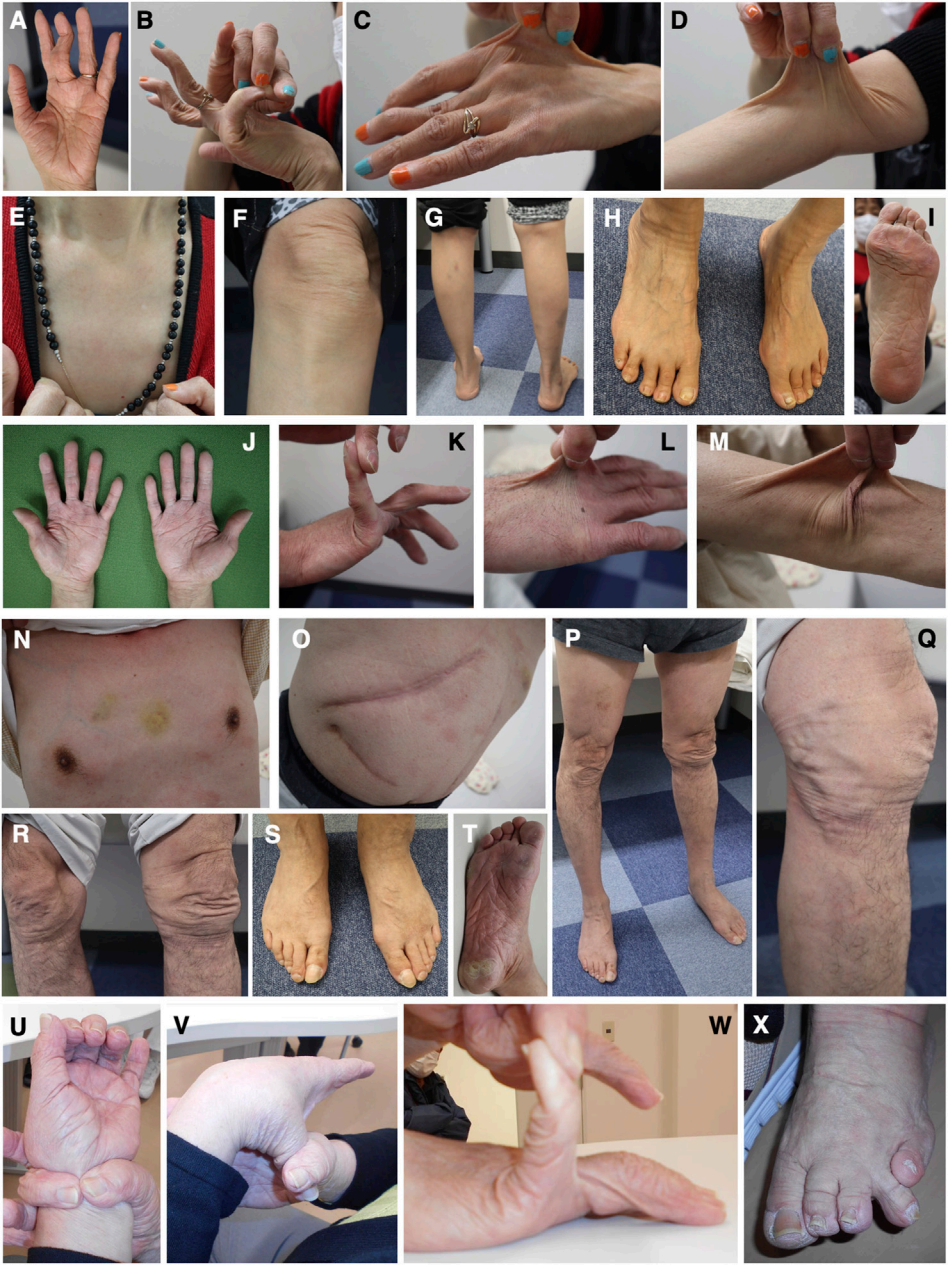
Clinical photographs of Patients 6, 7, and 9. **(A–I)** Patient 6 at age 59 years, showing wrinkled palms **(A)**, hypermobile finger joints **(B)**, skin hyperextensibility, translucency, and bruisability **(C–G)**, mildly atrophic thighs **(G)**, pes planus **(H)**, and moderate calluses on the soles **(I)**. **(J–T)** Patient 7 at age 61 years, showing wrinkled palms **(J)**, mild joint hypermobility with a Beighton score of 4/9 **(K)**, hyperextensible, bruisable, thin, velvety, and translucent skin **(L–N,R)**, without fragility or atrophic scars **(O)**, mild pectus excavatum **(N)**, atrophic thighs **(P)**, multiple varices at the knees **(Q)**, pes planus **(S)**, and severe calluses on the soles **(T)**. **(U–X)** Patient 9 at age 69 years, showing brachydactyly with excessive skin **(U,W)** and toe deformities **(X)**, but no generalized joint hypermobility **(V,W)**.

Patient 6 is a 59-year-old woman whose parents are Chinese and cousins. Part of her case information has been described recently (Ishiguro et al., 2022). She had shoulder dislocation at age 1 year, and was readily bruisable and experienced recurrent dislocation of the hip and finger joints, and repetitive episodes of anal prolapse, in her preschool days. She developed knee dislocation when she sat on her heels in her elementary school days. At the delivery of her second child and at surgeries for appendicitis and breast cancer, she experienced massive bleeding. At age 49 years, she was referred to a genetics clinic with suspected EDS based on bruisability, skin hyperextensibility, and recurrent dislocation. While her skin hyperextensibility was mild, she had multiple bruises that included the soles of her feet, and reported massive swelling after insect bites. She had dislocation of the fingers, toes, and shoulders. Cardiac ultrasonography detected no abnormalities in the wall motion and mitral valves, and no dilatation of the aortic root. Her condition was complicated by generalized and wandering pain, sometimes unrelated to joint dislocation or other physical manifestations, and was successfully treated with duroxetine. At age 58 years, she developed anal laceration and rectal hematoma requiring emergency surgery. When seen by us at age 59 years, she had wrinkled palms (Figure 5A) and skin hyperextensibility, translucency, and bruisability (Figures 5C–G), and hypermobile finger joints (Figure 5B), mildly atrophic thighs (Figure 5G), pes planus (Figure 5H), and moderate calluses on the soles (Figure 5I).

Modified NGS panel analysis using long PCR-amplified product detected homozygous c.12150C>G,p.(Arg4050 = ) and c.12174C>G,p.(Cys4058Trp) variants in exon 40, which were confirmed by Sanger sequencing (Figure 3Fa). MLPA showed a normal copy of *TNXB* and *CYP21A2* (data not shown). Therefore, this patient was determined to have a type 2 gene conversion (Figure 1B). Western blot analysis showed complete absence of sTNX (Figure 3Fb).

### Patient 7

Patient 7 is a 61-year-old Japanese man whose parents were allegedly consanguineous. He was a low-birth-weight baby who was admitted to hospital in infancy because of digestive impairment, but showed no developmental delay. From early childhood, he showed skin hyperextensibility but not fragility, and at age 3 years began suffering from recurrent shoulder dislocation. He did not participate in physical exercise in his elementary school days. At age 10 years, he developed a perforation in the intestine caused by an abdominal bruise, and was treated with emergency surgery. He worked as a carpenter, but could not carry heavy objects and became a school janitor. He got married at age 25 years. He developed a perforation in the esophagus at age 27 years and was treated with emergency surgery and subsequent mechanical ventilation for a month. During this admission, he was suspected to have EDS. He showed dilatation of the bladder resulting in difficulty with urination, and was managed with clean intermittent catheterization (CIC). He underwent a surgical reduction of the bladder that provided only tentative relief, and CIC was reintroduced. At age 42 years, he had angina pectoris. At age 57 years, he developed an inguinal hernia and diverticulitis, and was found to have bladder diverticula. When seen by us at age 61 years, his height was 167.3 cm (−0.6SD), weight was 60.0 kg (−0.3SD), OFC was 55.0 cm (−1.5SD), and arm span was 172.5 cm. He had wrinkled palms (Figure 5J), mild joint hypermobility with a Beighton score of 4/9 (Figure 5K), skin that was hyperextensible, bruisable, thin, velvety, and translucent (Figures 5L–N, R), without fragility or atrophic scars (Figure 5O). He also had, mild pectus excavatum (Figure 5N), atrophic thighs (Figure 5P), multiple varices at the knees (Figure 5Q), pes planus (Figure 5S), severe calluses on the soles (Figure 5T), mild scoliosis with a Cobb angle of 14°, a high palate with crowded teeth.

Modified NGS panel analysis using long PCR-amplified product detected a heterozygous *TNXA*-derived 120-bp deletion in exon 35–intron 35 (c.11435_11524 + 30del), c.12150C>G,p.(Arg4050 = ) and c.12174C>G,p.(Cys4058Trp) in exon 40, c.12204 + 39dup and c.12204 + 43T>G in intron 40, and c.12628-52A>G in intron 43. The four variants in exon 40 and intron 40 were confirmed by Sanger sequencing (data not shown). MLPA showed the one-copy loss of exon 35 in *TNXB* and a one-copy loss of *CYP21A2* (Figure 3Ga). Western blot analysis showed complete absence of sTNX (Figure 3Gb). Long-PCR using primers TNXB-ex35-F and TNXB-ex44-R, which specifically amplify the normal allele of *TNXB* exon 35 (i.e., not the exon 35 deletion allele), and Sanger sequencing confirmed the presence of the four variants in exon 40 and intron 40 (Figure 3Gc). Therefore, it was determined that Patient 7 was compound heterozygous for a type 1 gene conversion and a type 2 *TNXB/TNXA* fusion gene (Figure 1B).

### Patient 8

Patient 8 is an 8-year-old Japanese girl. Her father has skin hyperextensibility, joint hypermobility, and joint pain. Her mother, who has skin hyperextensibility and translucency, experienced fractures in the shoulder, arm, and foot. Her older brother had Perthes disease. She was born by cesarean section at 38 weeks and 4 days of gestation after a pregnancy complicated by polyhydramnios. Her birth weight was 3850 g (+2.7SD), length was 50.1 cm (+0.9SD), and OFC was 37.8 cm (+3.1SD). At age 1 month, generalized joint hypermobility was noticed, and she began receiving physical therapy from age 4 months. In infancy, she sucked poorly and had constipation requiring swab bougienage. At age 1 year, she was suspected to have autism spectrum disorder based on excessive stranger anxiety. When seen by us at age 3 years and 7 months, she was falling down easily and had generalized joint hypermobility with a Beighton score of 8/9, pes planovalgus, mild tonsil hypertrophy, a slender uvula, mild tooth irregularity, and mild reversed occlusion, but no high palate. At age 6 years and 5 months, an epiphysis of her ankle was fractured, and at age 8 years and 2 months, her weight was 24.8 kg (−0.2SD), height was 126.8 cm (+0.2SD), and OFC was 54.5 cm (+1.7SD). She frequently had stomatitis, peripheral cyanotic skin in a cool environment, and hyperventilation. Her Beighton score was 9/9. She showed limited extension of the interphalangeal joints in bilateral thumbs, and limited flexion and trigger finger of the left 4th finger, with morning stiffness. She had skin hyperextensibility and bruisability, but no episodes of skin laceration requiring surgical suture, sprains, or dislocations.

Standard NGS panel analysis detected a heterozygous nonsense variant c.8585G>A,p.(Trp2862*) in exon 25, which was confirmed by Sanger sequencing (Figure 3H, left). Modified NGS panel analysis using long PCR-amplified product detected a heterozygous *TNXA*-derived 120-bp deletion in exon 35–intron 35 (c.11435_11524 + 30del). MLPA showed the one-copy loss of exon 35 in *TNXB* and a normal copy of *CYP21A2* (type 1 gene conversion) (Figure 3H, right). Sanger sequencing and MLPA analysis were performed on her parents, revealing the heterozygous nonsense variant in her mother and the one-copy loss of exon 35 in *TNXB* in her father (Figure 3H). Therefore, it was determined that Patient 8 was compound heterozygous for the nonsense variant and a type 1 gene conversion (Figure 1B).

### Patient 9

Patient 9 is a 69-year-old Japanese woman. Recurrent dislocation of the right shoulder that could be treated by self-reposition began at age 6 years and continued into adulthood. She frequently had subcutaneous hematomas, one of which led to her being suspected of having EDS in her 30s. At age 58 years, she developed a diaphragmatic hernia (hiatal hernia) that was treated by surgery, followed by an abdominal hernia. She had a pulmonary embolism after surgery for gallstones. At age 65 years, vaginal prolapse, accompanied by difficulties in defecation and urination, was noticed. She was referred to us at age 69 years for genetic evaluation in view of surgery for progressive vaginal prolapse. Brachydactyly with excessive skin (Figures 5U, W) and toe deformities (Figure 5X) were observed, but generalized joint hypermobility was not noted (Figures 5V, W). The hiatal and abdominal hernias were also found to be progressive. After this referral, she developed a small bowel perforation that healed spontaneously.

Standard NGS panel analysis detected a heterozygous frameshift variant c.9271dup,p.(Gln3091Profs*31) in exon 27, which was confirmed by Sanger sequencing (Figures 3A–I, left). Modified NGS panel analysis using long PCR-amplified product detected a heterozygous *TNXA*-derived 120-bp deletion in exon 35–intron 35 (c.11435_11524 + 30del), c.12150C>G,p.(Arg4050 = ) and c.12174C>Gp.(Cys4058Trp) in exon 40, c.12204 + 39dup and c.12204 + 43T>G in intron 40, and c.12628-52A>G in intron 43. The four variants in exon 40 and intron 40 were confirmed by Sanger sequencing (data not shown). MLPA showed the one-copy loss of exon 35 in *TNXB* and a one-copy loss of *CYP21A2* (type 1 *TNXB/TNXA* fusion gene) (Figure 3Ia, right). Long-PCR using primers TNXB-ex26-F and TNXB-ex35-R, which specifically amplify the normal allele of *TNXB* exon 35 (i.e., not the exon 35 deletion allele) and Sanger sequencing confirmed the presence of the frameshift variant in exon 27 (Figure 3Ib). Therefore, it was determined that Patient 9 was compound heterozygous for the frameshift variant and a type 1 *TNXB/TNXA* fusion gene (Figure 1B).

## Discussion

We have described detailed clinical and molecular findings of nine unrelated patients with clEDS who were found to have biallelic *TNXB* variants. This is the first report to apply an NGS-based method to screen for *TNXB* variants and *TNXA*-derived sequences recombined into *TNXB*, and represents the third largest cohort of clEDS patients*.*

Hyperextensible skin, recurrent dislocations, easily bruisable skin, and hand and foot deformities were observed in >80% of the cases with available data. Eye bleeding, which is not included in the diagnostic criteria, was observed in three patients. Gastrointestinal complications were observed in the eight patients whose data were available, and included perforation in three patients, diverticulitis in three, gastrointestinal bleeding in two, intestinal obstruction in two, rectal or anal prolapse in four, and previously unreported gallstones in three. Clinical features of the current cohort (66.7% female; median age, 60 years) were compared with those of the largest cohort (Green et al., 2020) and the second largest cohort (Demirdas et al., 2017). Among the 20 patients reported by Green et al. (2020), 19 patients from 15 families had biallelic *TNXB* variants (68.4% female; median age, 34 years). Among the 17 patients reported by Demirdas et al. (2017), 14 patients from 11 families had biallelic *TNXB* variants (64.3% female; median age, 28 years). Eye bleeding was observed in four patients and two patients of the Green et al. and Demirdas et al. cohorts, respectively. Gastrointestinal complications, for which only rectal prolapse is included in the diagnostic criteria, were observed in six patients in Green et al. (2020), and comprised rupture in three patients, diverticulitis in three, gastrointestinal bleeding in two, and rectal prolapse in two, with no intestinal obstruction or gallstones. In the Demirdas et al. (2017) cohort, three patients had diverticulitis, one had gastrointestinal bleeding, and two had rectal prolapse, with no rupture or perforation, intestinal obstruction, or gallstones. The range of such events across all three cohorts (rupture/perforation in six patients, diverticulitis in nine, bleeding in five, obstruction in four, rectal/anal prolapse in six, and gallstones in three) highlight the importance and variability of gastrointestinal complications in patients with clEDS. The higher frequency of gastrointestinal complications in the current cohort might be a reflection of the higher median age compared to the other cohorts. This higher median age population in the current cohort could be useful in detecting age-dependent manifestations such as gastrointestinal complications, but could be less beneficial in accurately describing childhood manifestations due to recall bias.

To date, biallelic variation in *TNXB* has been documented in 50 clEDS patients from 43 families. *TNXA*-derived variations were detected in 77.8% (7/9 families) in the current cohort and in 76.7% (33/43 families) in the previous study. Most of the previously reported *TNXB* variants are null variants and include the 120-bp deletion, nonsense variants, frameshift variants, and splice site variants, which are predicted to lead to nonsense-mediated mRNA decay. In the Schalkwijk et al. (2001) study using Western blot analysis of serum samples and patient-derived fibroblast-conditioned medium, no TNX was detected in five patients with an EDS phenotype. In a type 2 *TNXB/TNXA* fusion or a type 2 gene conversion, the missense variant c.12174C>G,p.(Cys4058Trp) is considered to be pathogenic, but the effect of the altered protein is unknown. A homozygous missense variant c.12174C>G (i.e., either type 2 *TNXB/TNXA* fusion gene or type 2 gene conversion in both alleles) has been reported in multiple patients, including one patient by Hendriks et al. (2012)/Demirdas et al. (2017), one by Chen et al. (2016), and seven by Green et al. (2020). In the patient described by Hendriks et al. (2012)/Demirdas et al. (2017), there was a complete absence of serum TNX on Western blot analysis (Supplementary Table S3). In the current study, Patient 6 also showed a complete absence of serum TNX on Western blot analysis (Figure 3Fb). A compound heterozygous c.12174C>G with another variant (i.e., either type 2 *TNXB/TNXA* fusion gene or type 2 gene conversion in one allele) has also been reported in multiple patients, including one patient by Schalkwijk et al. (2001)/Demirdas et al. (2017). These studies reported a complete absence of TNX in serum and fibroblast-conditioned medium (Supplementary Table S3). In view of all these findings, the main effect of this missense variant is likely to be a disturbance in secretion or susceptibility to degradation of TNX, rather than a defect in production. In conclusion, we developed an NGS-based screening system that can accurately detect *TNXB* variants and *TNXA*-derived sequences recombined into *TNXB*. *TNXA*-derived variations were found in >75% of the current cohort, comparable to previous reports. Gastrointestinal complications (e.g., perforation, diverticulitis, gastrointestinal bleeding, intestinal obstruction, rectal/anal prolapse, and gallstones) were observed particularly frequently in the current cohort, highlighting the importance of increasing awareness of the risk of gastrointestinal complications in clEDS.

## Data Availability

Original datasets are available in a publicly accessible repository. The mass spectrometry proteomics data have been deposited to the ProteomeXchange Consortium via the PRIDE (https://proteomecentral.proteomexchange.org/cgi/GetDataset) partner repository with the dataset identifier PXD043691. The variant data have been deposited to the Global Variome shared LOVD (https://databases.lovd.nl/shared/genes/TNXB) with the individual ID 435339, 435340, 435341, 435342, 435344, 435346, 435348, 435349, 435351.
