# Supplementary material for: Clinical and molecular delineation of classical-like Ehlers–Danlos syndrome through a comprehensive next-generation sequencing-based screening system
Source: Front Genet. 2023 Aug 30;14:1234804. doi: 10.3389/fgene.2023.1234804 (PMC10498456; doi:10.3389/fgene.2023.1234804)
Supplement: Supplementary file 1 [file Table1.DOCX]

**Supplementary Table S1. Ion AmpliSeq custom panel for 53 genes associated with Ehlers–Danlos syndromes and other hereditary connective tissue disorders**

| **Disorders** | **Genes** |
| --- | --- |
| Ehlers–Danlos syndrome | *ADAMTS2*, *AEBP1*, *B3GALT6*, *B4GALT7*, *C1R*, *C1S*, *CHST14*, *COL1A1*, *COL1A2*, *COL3A1*, *COL5A1*, *COL5A2*, *COL12A1*, *DSE*, *FKBP14*, *PLOD1*, *PRDM5*, *SLC39A13*, *TNXB*, *TNXA*, *ZNF469* |
| Marfan syndrome | *FBN1* |
| Loeys–Dietz syndrome | *SMAD2*, *SMAD3*, *TGFB2*, *TGFB3*, *TGFBR1*, *TGFBR2* |
| Familial thoracic aortic aneurysms and aortic dissections | *ACTA2*, *COL3A1*, *FBN1*, *LOX*, *MYH11*, *MYLK*, *SMAD3*, *TGFB2*, *TGFBR1*, *TGFBR2* |
| Arterial tortuosity syndrome | *SLC2A10* |
| Beals syndrome | *FBN2* |
| Shprintzen–Goldberg syndrome | *SKI* |
| Filamin A (FLNA)-related periventricular nodular heterotopia/otopalatodigital syndrome | *FLNA* |
| Ectopia lentis | *ADAMTSL4* |
| Osteogenesis imperfecta | *BMP1*, *COL1A1*, *COL1A2*, *CRTAP*, *FKBP10*, *IFITM5*, *LEPRE1*, *PLOD2*, *PPIB*, *SEC24D*, *SERPINF1*, *SERPINH1*, *SP7*, *TMEM38B*, *WNT1* |
| Osler disease | *ACVRL1*, *ENG*, *SMAD4* |
