# Supplementary material for: Clinical and molecular delineation of classical-like Ehlers–Danlos syndrome through a comprehensive next-generation sequencing-based screening system
Source: Front Genet. 2023 Aug 30;14:1234804. doi: 10.3389/fgene.2023.1234804 (PMC10498456; doi:10.3389/fgene.2023.1234804)
Supplement: Supplementary file 2 [file Table2.DOCX]

Supplementary Table S2. List of hg19 positions in a hotspot file, patterns of variant calls in NGS panel analysis, and MLPA results

|  |  | Position  (hg19) | *TNXB*  wild type | *TNXB/TNXA* fusion (type 1) | | *TNXB/TNXA* fusion (type 2) | | Gene conversion  (type 1) | | Gene conversion  (type 2) | |
| --- | --- | --- | --- | --- | --- | --- | --- | --- | --- | --- | --- |
|  |  |  |  | het | hom | het | hom | het | hom | het | hom |
| NGS | Normal exon 35 | chr6:32011599 | 0/0 | 0/0 | No Call | 0/0 | 0/0 | 0/0 | No Call | 0/0 | 0/0 |
|  | *TNXA*-derived 120-bp deletion^a^ | chr6:31978785 | No Call | 0/0 | 0/0 | No Call | No Call | 0/0 | 0/0 | No Call | No Call |
|  | c.12150C>G (exon 40)^b^ | chr6:32010286 | 0/0 | 0/1 | 1/1 | 0/1 | 1/1 | 0/0 | 0/0 | 0/1 | 1/1 |
|  | c.12174C>G (exon 40)^c^ | chr6:32010262 | 0/0 | 0/1 | 1/1 | 0/1 | 1/1 | 0/0 | 0/0 | 0/1 | 1/1 |
|  | c.12204+39dup (intron 40)^d^ | chr6:32010192 | 0/0 | 0/1 | 1/1 | 0/1 | 1/1 | 0/0 | 0/0 | 0/0 | 0/0 |
|  | c.12204+43T>G (intron 40)^e^ | chr6:32010189 | 0/0 | 0/1 | 1/1 | 0/1 | 1/1 | 0/0 | 0/0 | 0/0 | 0/0 |
|  | c.12628-52A>G (intron 43)^f^ | chr6:32009279 | 0/0 | 0/1 | 1/1 | 0/1 | 1/1 | 0/0 | 0/0 | 0/0 | 0/0 |
| MLPA | *TNXB* exon 35 | - | Normal | 1-copy  loss | 2-copy  loss | Normal | Normal | 1-copy  loss | 2-copy  loss | Normal | Normal |
|  | *CYP21A2* ^g^ | - | Normal | 1-copy loss | 2-copy  loss | 1-copy  loss | 2-copy  loss | Normal | Normal | Normal | Normal |

^a-g^Letters correspond to variant positions in the gene map illustrated in Figure 1B. het: heterozygous variant; hom: homozygous variant; No call: lack of coverage; 0/0: homozygous reference allele; 0/1: heterozygous carrying both reference and alternative alleles; 1/1: homozygous alternative allele.
