# Supplementary material for: Clinical and molecular delineation of classical-like Ehlers–Danlos syndrome through a comprehensive next-generation sequencing-based screening system
Source: Front Genet. 2023 Aug 30;14:1234804. doi: 10.3389/fgene.2023.1234804 (PMC10498456; doi:10.3389/fgene.2023.1234804)
Supplement: Supplementary file 3 [file Table3.DOCX]

Supplementary Table S3. Effects of the missense variant NM_019105.6:c.12174C>G in patients with clEDS

|  |  |  | Allele 1 | Allele 2 | Tenascin-X |
| --- | --- | --- | --- | --- | --- |
| c.12174C>G in both alleles | | | | | |
| This study | － | Patient 6 | Gene conversion (type 2) | Gene conversion (type 2) | Absence in serum |
| Hendriks et al. (2012) | － | Patient 1 | *TNXB/TNXA* fusion gene (type 2) | Gene conversion (type 2) | Absence in serum |
| & Demirdas et al. (2017) | － | Family X |  |  |  |
| Chen et al. (2016) | － | Patient 3 | *TNXB/TNXA* fusion gene (type 2) | *TNXB/TNXA* fusion gene (type 2) | N/A |
| Green et al. (2020) | － | Patient 6 | Gene conversion (type 2) | Gene conversion (type 2) | N/A |
|  | － | Patient 10 | Gene conversion (type 2) | Gene conversion (type 2) | N/A |
|  | － | Patient 11 | Gene conversion (type 2) | Gene conversion (type 2) | N/A |
|  | － | Patient 12 | Gene conversion (type 2) | Gene conversion (type 2) | N/A |
|  | － | Patient 17 | Gene conversion (type 2) | Gene conversion (type 2) | N/A |
|  | － | Patient 16 | *TNXB/TNXA* fusion gene (type 2) | *TNXB/TNXA* fusion gene (type 2) | N/A |
|  | － | Patient 18 | *TNXB/TNXA* fusion gene (type 2) | Gene conversion (type 2) | N/A |
| c.12174C>G in one allele | | | | | |
| This study | － | Patient 7 | *TNXB/TNXA* fusion gene (type 2) | Gene conversion (type 1) | N/A |
| Schalkwijk et al. (2001) | － | Family 2 | Gene conversion (type 2) | Gene conversion (type 1) | Absence in serum and fibroblast-conditioned medium |
| & Demirdas et al. (2017) | － | Family II |  |  |  |
| Green et al. (2020) | － | Patient 1 | Gene conversion (type 2) | Gene conversion (type 1) | N/A |
|  | － | Patient 19 | Gene conversion (type 2) | Gene conversion (type 1) | N/A |

N/A: not available.
